# MicroRNA-106a regulates autophagy-related cell death and EMT by targeting TP53INP1 in lung cancer with bone metastasis

**DOI:** 10.1038/s41419-021-04324-0

**Published:** 2021-10-30

**Authors:** Lei Han, Zeyong Huang, Yan Liu, Lijuan Ye, Dongqi Li, Zhihong Yao, Cao Wang, Ya Zhang, Hang Yang, Zunxian Tan, Jiadai Tang, Zuozhang Yang

**Affiliations:** 1 Bone and Soft Tissue Tumors Research Center of Yunnan Province, Department of Orthopaedics, Yunnan Cancer Hospital, The Third Affiliated Hospital of Kunming Medical University, Yunnan Cancer Center, Kunming, Yunnan China; 2 Medical School, Kunming University of Science and Technology, Kunming, Yunnan China; 3 Department of Pathology, Yunnan Cancer Hospital, The Third Affiliated Hospital of Kunming Medical University, Yunnan Cancer Center, Kunming, Yunnan China; 4 Department of Rehabilitation Medicine, The Second Affiliated Hospital of Kunming Medical University, Kunming, Yunnan China; 5 Department of Gastrointestinal Oncology, Yunnan Cancer Hospital, The Third Affiliated Hospital of Kunming Medical University, Yunnan Cancer Center, Kunming, Yunnan China

**Keywords:** Bone metastases, Non-coding RNAs

## Abstract

Bone metastasis is one of the most serious complications in lung cancer patients. MicroRNAs (miRNAs) play important roles in tumour development, progression and metastasis. A previous study showed that miR-106a is highly expressed in the tissues of lung adenocarcinoma with bone metastasis, but its mechanism remains unclear. In this study, we showed that miR-106a expression is dramatically increased in lung cancer patients with bone metastasis (BM) by immunohistochemical analysis. MiR-106a promoted A549 and SPC-A1 cell proliferation, migration and invasion in vitro. The results of bioluminescence imaging (BLI), micro-CT and X-ray demonstrated that miR-106a promoted bone metastasis of lung adenocarcinoma in vivo. Mechanistic investigations revealed that miR-106a upregulation promoted metastasis by targeting tumour protein 53-induced nuclear protein 1 (TP53INP1)-mediated metastatic progression, including cell migration, autophagy-dependent death and epithelial–mesenchymal transition (EMT). Notably, autophagy partially attenuated the effects of miR-106a on promoting bone metastasis in lung adenocarcinoma. These findings demonstrated that restoring the expression of TP53INP1 by silencing miR-106a may be a novel therapeutic strategy for bone metastatic in lung adenocarcinoma.

## Introduction

Lung cancer has the highest morbidity and mortality rate in the world. Approximately 25% of cancer-related deaths are caused by lung cancer. In most countries, the incidence and mortality of lung cancer are showing a significantly increasing trend [1]. Approximately 30–40% of lung cancer patients eventually develop bone metastasis (BM), but the treatment of these patients is extremely poor [2]. Moreover, BM brings persistent pain to the patient and increases the risk of fracture, seriously affecting the quality of life of the patient [3, 4]. Therefore, fully understanding the signalling networks involved in BM of lung cancer is essential for the development of novel anti-metastatic strategies.

MicroRNAs (miRNAs) are widely occurring noncoding RNAs of approximately 19–22 nucleotides in size and pivotal in the regulation of multiple cellular functions, including cell proliferation and migration [5, 6]. Additionally, the dysregulation of miRNA can lead to cancer formation and metastases. MiRNAs can function as potential therapeutic targets in the progression of BM in a variety of cancers, for example, miR-15/16/21/141/19a in prostate cancer [7–9], miR-131/429/203/30/218/124 in breast cancer [10–14] and miR-192/33/335 in lung cancer [15–17]. MiRNA-106a (miR-106a), a member of the miR-17 family, has been found to be aberrantly expressed in diverse types of cancer [18, 19] and is correlated with the occurrence and metastasis of cancer [20]. For instance, high expression of miR-106a in serum was positively associated with cancer stages and poor survival in lung adenocarcinoma [21]. The high expression of miR-106a in the serum is considered as a useful parameter in identifying the chemotherapy resistance [22–24]. This evidence implies that miR-106a severely restricts the effective treatment of lung cancer, especially in patients with advanced BMs. Therefore, it is urgent to develop effective anti-BM strategies to relieve adverse symptoms of bone-related events. In a previous study, we found that miR-106a was highly expressed in lung adenocarcinoma tissues with BM. However, the role and mechanism of miR-106a in lung adenocarcinoma with BM have not been elucidated.

As a complex and multistep process, BM involves diverse biological changes, such as epithelial–mesenchymal transition (EMT), tumour angiogenesis and development of the tumour microenvironment. Moreover, several types of programmed cell death, including autophagy, apoptosis and necroptosis, have been confirmed to be critical for metastasis in lung cancers. Here we demonstrated that miR-106a targets tumour protein 53-induced nuclear protein 1 (TP53INP1) to promote BM in lung adenocarcinoma by regulating autophagy and EMT. Our results suggested that targeting miR-106a/TP53INP1/autophagy signalling may represent a potential therapeutic strategy for lung adenocarcinoma with BM.

## Result

### MiR-106a is highly expressed in lung adenocarcinoma patients with BM and is associated with poor prognosis

**Fig. 1:**
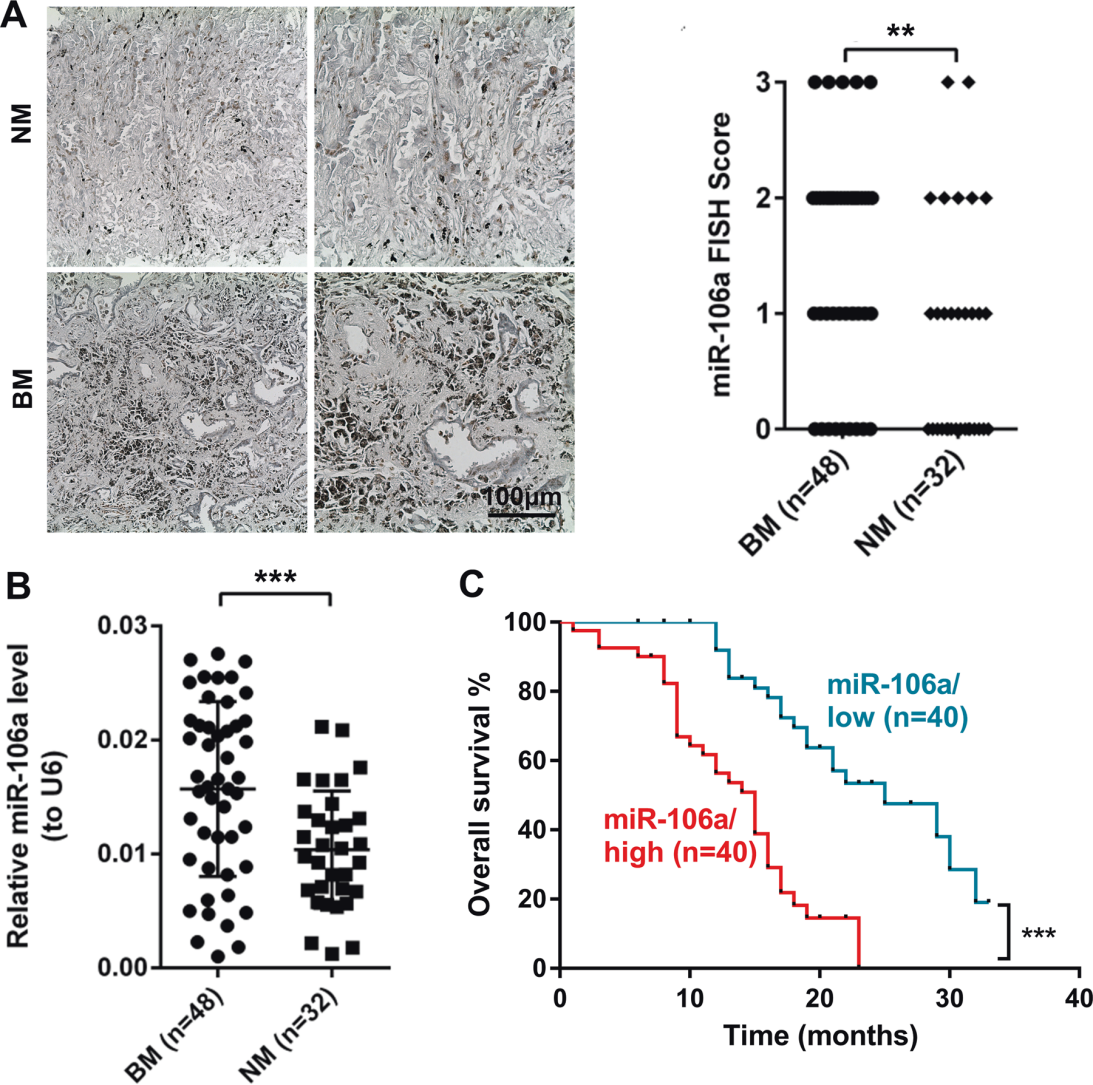
MiR-106a is highly expressed in the patients of lung adenocarcinoma with bone metastasis and associated with poor prognosis. MiR-106a expression level of miR-106a in the tissues of BM and NM with ISH (**A**) and qRT-PCR (**B**). **C** Kaplan–Meier analysis for the survival of lung adenocarcinoma patients with high or low expression levels of miR-106a. BM bone metastasis, NM non-metastasis.

Our previous study showed that miR-106a is one of the most pivotal BM-related miRNAs in lung adenocarcinoma by high-throughput sequencing [25]. Using in situ hybridization (ISH) (Fig. 1A) and quantitative reverse transcription polymerase chain reaction (qRT-PCR; Fig. 1B), we first confirmed the differential expression of miR-106a in lung adenocarcinoma tissues of 80 cases with or without BM. Compared to non-metastatic (NM) lung adenocarcinoma, the expression of miR-106a was remarkably upregulated in lung adenocarcinoma with BM, consistent with our previous research.

To determine the clinical significance of miR-106a in lung adenocarcinoma, we analysed the association between miR-106a expression and various lung adenocarcinoma clinicopathological parameters. Eighty patients with lung adenocarcinoma were divided into low/high miR-106a expression groups based on the median value of the qRT-PCR results. As shown in Table 1, the expression of miR-106a was significantly associated with BM but had no correlation with sex, age, differentiation degree or lymphatic metastasis. Survival analysis showed that non-small cell lung cancer (NSCLC) patients with high expression of miR-106a had a poorer prognosis (Fig. 1C). According to the results of the analysis on risk factors for overall survival in NSCLC patients, BM and high expression of miR-106a were identified as independent risk factors (Table S1).

**Table 1:** Correlation between clinicopathological features and the expression of miR-106a.

Characteristics	miR-106 expression		*P*
	Low (*n*, %)	High (*n*, %)	
Gender			
Male	25 (56.8%)	19 (43.2%)	0.178
Female	15 (41.7%)	21 (58.3%)	
Age (years)			
<54	23 (62.2%)	14 (37.8%)	0.044
≥54	17 (39.5%)	26 (60.5%)	
Differentiated degree			
High–middle differentiation	14 (41.2%)	20 (58.8%)	0.175
Low differentiation	26 (56.5%)	20 (43.5%)	
Lymphatic metastasis			
Yes	27 (51.9%)	25 (48.1%)	0.639
No	13 (46.4%)	15 (53.6%)	
Bone metastasis			
Yes	11 (22.9%)	37 (77.1%)	<0.001
No	29 (90.6%)	3 (9.4%)	

Taken together, these results indicated that upregulation of miR-106a may represent an independent risk factor affecting BM and the prognosis of patients with lung adenocarcinoma.

### MiR-106a enhances the metastatic capacity of lung adenocarcinoma in vivo and in vitro

**Fig. 2:**
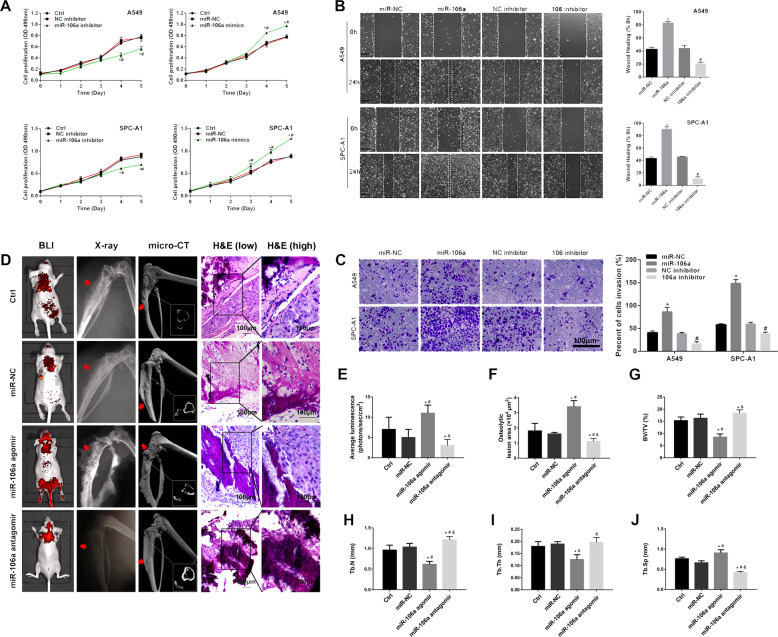
The effect of miR-106a on lung adenocarcinoma with bone metastasis in vivo and in vitro. **A** The proliferation of A549 and SPC-A1 cells by transfection of miR-106a mimics/inhibitor. *P < 0.05, compared with Ctrl; ^#^*P* < 0.05, compared with NC inhibitor/mimics. The effect of miR-106a on migration and invasion of A549 and SPC-A1 were measured by wound healing assay (**B**) and Transwell assay (**C**). *P < 0.05, miR-106a mimics compared with miR-NC; ^#^*P* < 0.05, miR-106a inhibitor compared with NC inhibitor. **D**–**J** The representative BLI, osteolytic lesion area on X-ray, micro-CT and H&E stain images were captured from the nude mice that had been treated with miR-106a agomir, antagomir or NC. Scale bars: 100 μm. *P < 0.05, compared with Ctrl; ^#^*P* < 0.05, compared with miR-NC; ^&^*P* < 0.05, compared with miR-106a agomir.

To explore the role of miR-106a in BM of lung adenocarcinoma, A549 and SPC-A1 lung cancer cell lines were transfected with miR-106a mimic, miR-106a inhibitor or miR-NC. Compared to miR-NC-transfected cells, the miR-106a mimic significantly enhanced A549 and SPC-A1 cell proliferation, while the miR-106a inhibitor had the opposite effect (Fig. 2A). Next, using wound healing and Transwell assays, we investigated the effect of miR-106a on lung adenocarcinoma cell migration and invasion. Results showed that cell monolayer restoration was induced in both A549 and SPC-A1 cells transfected with miR-106a mimics compared to scramble-transfected cells at 24 h (Fig. 2B). In addition, Transwell assays showed that the number of migratory cells significantly increased in A549 and SPC-A1 cells transfected with miR-106a mimic compared to miR-NC (Fig. 2C).

To determine the role of miR-106a in BM in vivo, gain-of-function studies were performed by injecting lung cancer cells transfected with 106a agomir into the heart’s left ventricle of nude mice. Bioluminescence imaging (BLI) showed that transfection of miR-106a agomir promoted the dissemination of cancer cells to other tissues, especially to the skeleton, while the transfection of miR-106a antagomir got the opposite results (Fig. 2D). X-ray, micro-computed tomography (micro-CT) and haematoxylin–eosin (H&E) staining were used to further determine the effect of miR-106a on bone destruction. As shown in Fig. 2E–J, significantly more BMs and bone destruction were observed in mice infected with miR-106a agomir, and miR-106a antagomir reduced BM and bone destruction compared to Ctrl.

These data indicated that miR-106a may enhance the BM capability of lung adenocarcinoma cells.

### Identification of TP53INP1 as the negative factor of miR-106a involved in lung cancer with BM

**Fig. 3:**
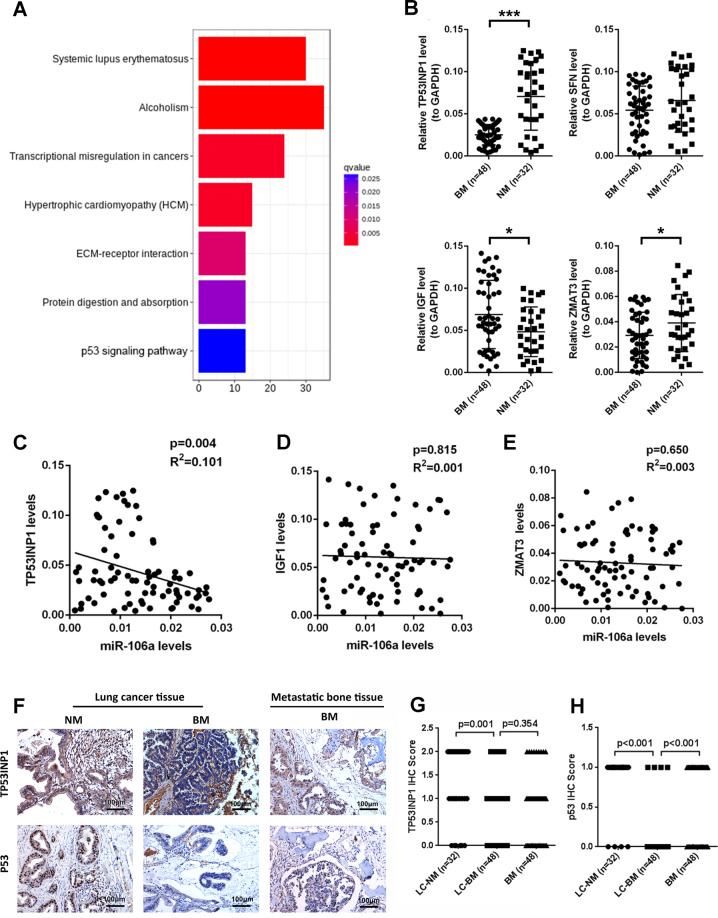
Identification of TP53INP1 as the negative factor of miR-106a involved in the lung cancer with bone metastasis. **A** RNA-seq analysis identified p53 pathway to be involved in miR-106a-mediated lung cancer bone metastasis. **B** TP53INP1, SFN, IGF1 and ZMAT3 expression in NM and BM patients. *P < 0.05; ***P < 0.001. **C**–**E** The correlation between TP53INP1/IGF-1/ZMAT3 and miR-106a expression in lung adenocarcinoma tissues. **F** Representative TP53INP1 and p53 immunohistochemical staining images of the lungs and bone tissue. **G**, **H** IHC scores representing TP53INP1 and p53expression. Data are means ± SD. *P < 0.05, unpaired two-tailed *t* test. Scale bars: 100 μm. LC lung cancer, NM non-metastasis, BM bone metastasis.

To elucidate the molecular mechanism by which miR-106a promotes lung cancer metastasis, RNA-seq was performed to identify the mRNA expression profile. The differentially expressed genes (DEGs) between miR-NC- and miR-106a-transfected cells were significantly enriched in the p53 signalling pathway (Figs. 3A and S1 and Tables S2 and S3). Next, we selected several differential genes involved in this pathway for verification, including TP53INP1, stratifin, insulin-like growth factor 1 (IGF1) and zinc finger matrin-type 3 (ZMAT3). The qPCR showed that these genes were differentially expressed between BM and NM (Fig. 3B). Among them, there was a negative correlation between TP53INP1 and miR-106a expression in lung adenocarcinoma tissues (Fig. 3C–E). Furthermore, lung adenocarcinoma sections and BM sections from 80 patients were performed for immunohistochemical (IHC) staining (Fig. 3F). Compared with the NM, p53 and TP53INP1 protein levels in the tissues of lung cancer with BM were 0.49- and 0.21-fold lower, respectively (Fig. 3G, H). The expression of miR-106a negatively correlated with TP53INP1, and they were both correlated with the presence of BMs (Table S4). These results supported that miR-106a/TP53INP1 is crucial for lung adenocarcinoma metastasis.

### The effect of miR-106a/TP53INP1 on metastatic progression of cells in vitro and in vivo

**Fig. 4:**
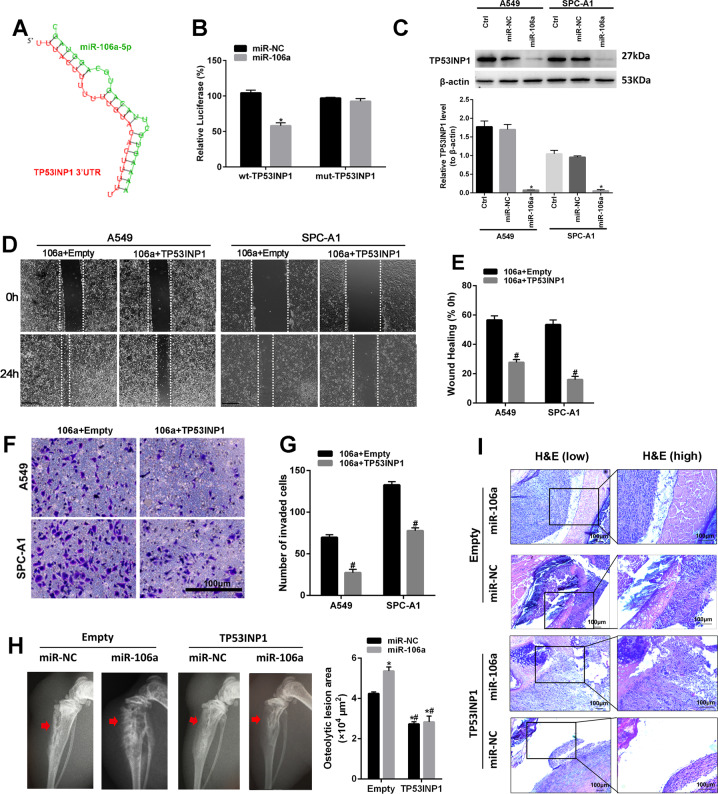
TP53INP1 was a target gene of miR-106a and was involved in metastatic progression. **A** The binding site between miR-106a and the 3’-UTR of TP53INP1 was predicted by TargetScan. **B** Luciferase reporter assay was performed to validate the interaction between miR106a with TP53INP1 3’-UTR. **C** The protein expression of TP53INP1 after transfection with miR-106a/miR-NC. **D**, **E** The role of miR-106a and TP53INP1 on the migration of A549 and SPC-A1 cells was detected by wound healing assay. Scale bars: 100 μm. **F**, **G** The role of miR-106a and TP53INP1 on the invasion of A549 and SPC-A1 cells was detected by Transwell assay. Scale bars: 100 μm. **H**, **I** The representative osteolytic lesion area on X-ray images and H&E stain were captured from the nude mice. *P < 0.05, compared with Empty + miR-NC; ^#^*P* < 0.05, compared with Empty + miR-106a.

Next, TargetScan was used to predict miR-106a target genes, and the 1217–1243 region in the TP53INP1 3’-untranslated region (UTR) was found exhibiting a high degree of complementarity with miR-106a (Fig. 4A). To validate the experimental computational data, the TP53INP1 3’-UTR was subcloned downstream of the luciferase open reading frame, and a dual-luciferase reporter assay was performed. Luciferase activity was markedly reduced only in the cells cotransfected with the miR-106a and the wild-type TP53INP1 3’-UTR, but not in cells cotransfected with the mutant TP53INP1 3’-UTR (Fig. 4B). Moreover, overexpression of miR-106a decreased TP53INP1 protein levels in lung cancer cells (Fig. 4C). These data demonstrated that TP53INP1 is a target of miR-106a.

We then measured the effect of miR-106a/TP53INP1 on the ability of cells to successfully navigate metastatic progression, including invasion of the basement membrane and cell migration, intravasation into the surrounding vasculature or lymphatic system and survival during circulation. SPC-A1 and A549 cells were transfected with the TP53INP1-overexpression plasmid (Fig. S2). In vitro, wound healing assays and Transwell assays demonstrated that overexpression of TP53INP1 significantly reversed the promoting effect of miR-106a on lung adenocarcinoma migration and invasion (Fig. 4D–G). In vivo, in the absence of TP53INP1, miR-106a-inducible cells generated BM after left heart ventricle injections in nude mice. After restoring the TP53INP1 expression, the tumour grew slowly, and none of the TP53INP1-treated mice developed BM (Fig. 4H, I). These data demonstrated miR-106a regulated lung adenocarcinoma BM through directly targeting TP53INP1.

### MiR-106a/TP53INP1 regulated apoptosis- and EMT-related pathway in vitro

**Fig. 5:**
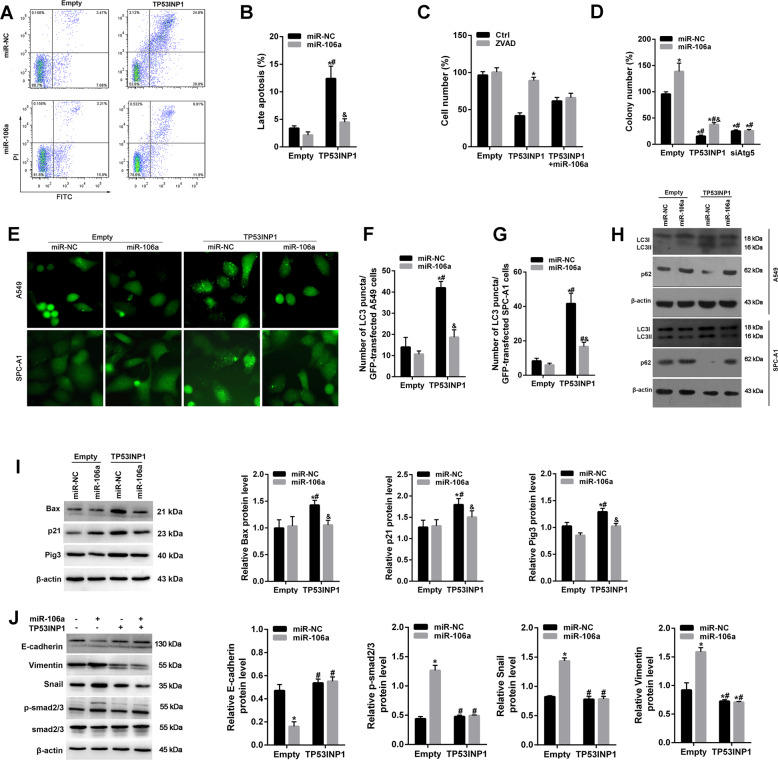
MiR-106a/TP53INP1 regulated apoptosis- and EMT-related pathway in vitro. **A**, **B** FACS analysis of cell apoptosis of SPC-A1 cells treated with miR-106a mimic and/TP53INP1. **C** Cell viability showed that the apoptosis of cells treated with TP53INP1 was inhibited in presence of a caspase inhibitor (Z-VAD-FMK). **D** Clonogenic assays showed that ATG5 knockdown abolished the effect of miR-106a in the number of colonies. miR-NC + Empty vector as control. **E**–**G** A549 and SPC-A1 cells were treated with pIRES2-EGFP-LC3 plasmid for 24 h. Then cells were observed in the fluorescence microscope. Scale bars: 20 μm. The number of GFP-LC3 puncta per cell was quantified and presented as mean ± SE from 100 randomly selected cells (*n* = 3). **H** Western blotting analysis of LC3 and p62 expression in A549 and SPC-A1 cells after transfected with miR-106a or/and TP53INP1. **I** Western blot analysis of the apoptosis-related protein expression of p21, Bax and Pig after transfected with miR-106a. **J** Western blot analysis of the expression of EMT-related protein, including E-cadherin, vimentin, snail, smad2/3 and p-smad2/3, after transfected with miR-106a mimics. *P < 0.05, compared with Empty + miR-NC; ^#^*P* < 0.05, compared with Empty + miR-106a; ^&^*P* < 0.05, compared with TP53INP1 + miR-NC.

Flow cytometric analysis showed that overexpression of TP53INP1 significantly increased the late apoptosis of SPC-A1 cells, but this effect was partially reversed by miR-106a (Fig. 5A, B). Moreover, Z-VAD-FMK, a caspase inhibitor, attenuated the inhibitory role of miR-106a in cell death induced by TP53INP1 overexpression, indicating that the inhibition of caspase-dependent apoptosis is critical for miR-106 to maintain tumour cell survival (Fig. 5C). In addition, ATG5 knockdown abolished the effect of miR-106a on the number of colonies (Fig. 5D). Furthermore, the transfection of miR-106a mimics alone did not affect the essential autophagy, but notably inhibition of TP53INP1 overexpression increased the number of GFP-LC3 fluorescent puncta of A549 and SPC-A1 cells (Fig. 5E–G). This result was confirmed by western blot analysis for the accumulation of LC3II and the decrease of p62 (Fig. 5H). In addition, miR-106a/TP53INP1 regulated the expression of several pro-apoptotic genes, p21, Bax and Pig3 (Fig. 5I). These data suggested that miR-106a could inhibit the autophagic-dependent cell death induced by TP53INP1.

EMT was the leading explanation for how and why metastasis happens. Thus we examined the effect of miR-106a/TP53INP1 on EMT by western blot analysis. The results showed that miR-106a decreased E-cadherin levels and increased the levels of vimentin and snail. Moreover, miR-106a enhanced the phosphorylation levels of smad2/3, which is pivotal for EMT (Fig. 5J). These effects on miR-106a can be blocked by the upregulation of TP53INP1.

### The pro-metastatic role of miR-106a is partly dependent on autophagy

TP53INP1-induced cell death is autophagy dependent [26]. Another study reported that autophagy inhibition could promote EMT in alveolar epithelial cells, which contributes to the distant metastasis of tumours [27, 28]. It indicates that autophagy might play an important role in miR-106a/TP53INP1 regulation of BM in lung cancer. Thus, we further investigated the role of autophagy in the process by which miR-106a promotes BM in lung cancer.

**Fig. 6:**
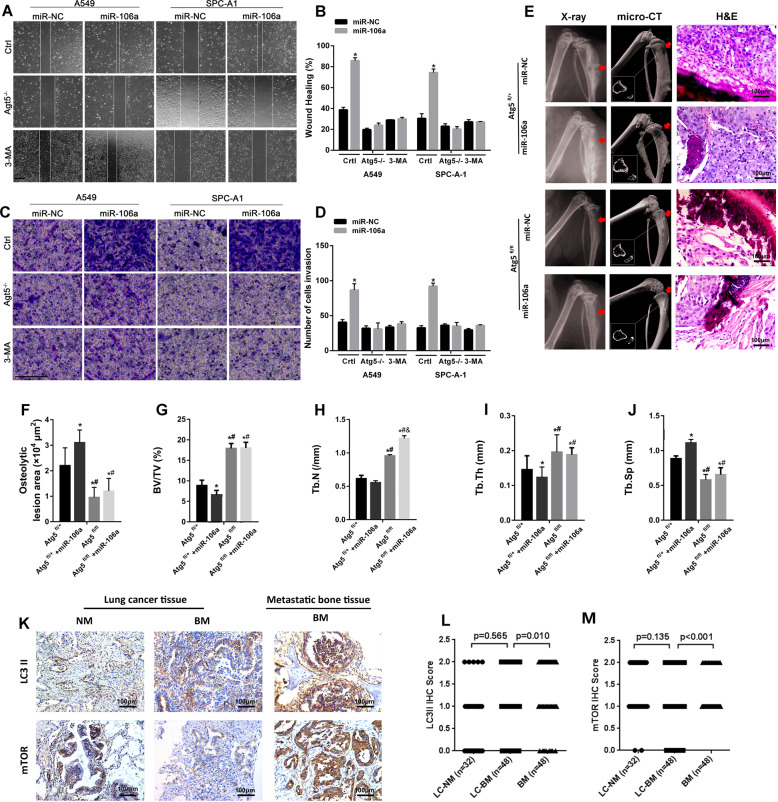
The pro-metastasis role of miR-106a is partly dependent on autophagy. **A**, **B** Wound healing analysis of the migration capability of A549 and SPC-A1 cells after treatment with siATG5 or 3-MA; *P < 0.05, compared with siATG5 + miR-106a or 3-MA + miR-106a. **C**, **D** Transwell migration of A549 and SPC-A1 cells were analysed under a microscope after treatment with siATG5 or 3-MA; *P < 0.05, compared with siATG5 + miR-106a or 3-MA + miR-106a. **E**–**J** Representative images of osteolytic lesion area on X-ray, micro-CT scans and H&E stain images from ATG5^fl/fl^ or ATG5^fl/+^ nude mice that had been injected with SPC-A1 and treated with miR-106a agomir or miR-NC; *P < 0.05, compared to Atg5^fl/+^; ^#^*P* < 0.05, compared to Atg5^fl/+^ + miR-106a; ^&^*P* < 0.05, compared to Atg5^fl/+^ + miR-106a. **K**, **L** Representative LC3II and mTOR immunohistochemical staining images of the lungs and bone tissue. **M** IHC scores representing LC3II and mTOR expression. Data are means ± SD. *P < 0.05, unpaired two-tailed *t* test. Scale bars: 100 μm. LC lung cancer, NM non-metastasis, BM bone metastasis.

Atg5 functional loss and the autophagy inhibitor 3-MA were used to inhibit cellular autophagy in A549 and SPC-A1 cells. The migration and invasion of lung adenocarcinoma cells promoted by miR-106a were significantly attenuated by Atg5 short hairpin RNA or 3-MA, which indicated that the pro-metastatic role of miR-106a was partially dependent on autophagy (Fig. 6A–D). Next, we investigated the role of autophagy in miR-106a-induced BM in vivo. As shown in Fig. 6E–J, the X-ray, micro-CT and H&E staining assay revealed that miR-106a dramatically increased BM burden in Atg5^fl/+^ nude mice inoculated with miR-106-overexpressing SPC-A1 cells.

Additionally, we used IHC staining to determine whether autophagy-related protein levels in samples from lung tumour patients predicted a greater rate of metastasis (Fig. 6K). There was no significant difference in the expression of LC3 or mammalian target of rapamycin (mTOR) in lung tissue from BM and NM patients (Fig. 6L, M). However, the expression levels of LC3 and mTOR in the metastatic bone tissues were higher than primary lung adenocarcinoma tissues. These data indicated miR-106a promoting BM in lung adenocarcinoma partly depended on autophagy.

## Discussion

**Fig. 7:**
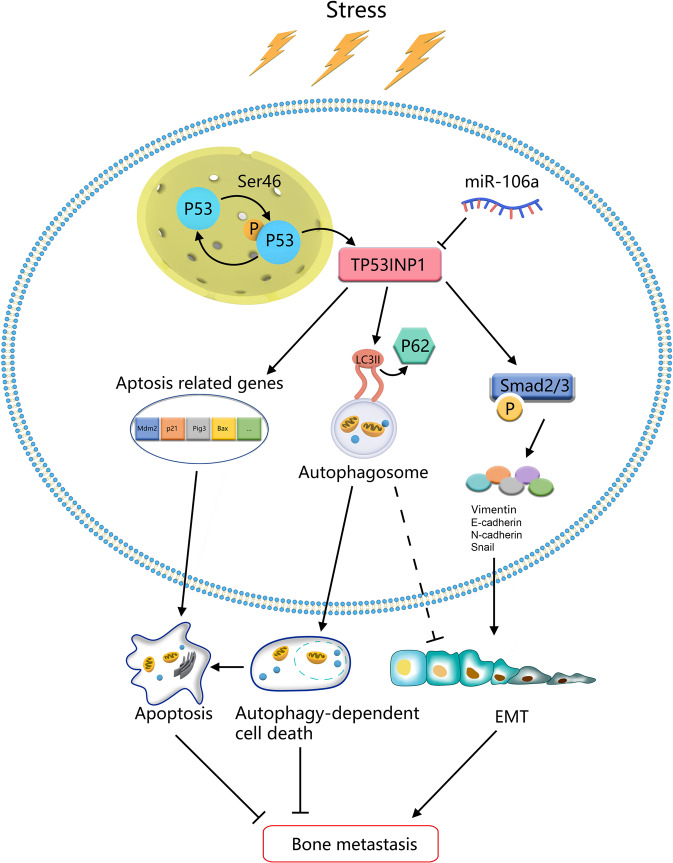
Schematic overview of the role of miR106a in lung cancer with bone metastasis. In response to diverse stresses, p53 was phosphorylated at Ser-46 and activate TP53INP1 signalling, which was involved in the cellular physiological process through a feedback loop: the phosphorylation of p53 at Ser-46 regulates TP53INP1 expression, and TP53INP3 regulates p53 activity via increasing the phosphorylation level of p53 at Ser-46. Subsequently, TP53INP1 could affect lung adenocarcinoma bone metastasis through three different ways: (1) TP53INP1 affects the expression of downstream target genes of p53, including p21, Pig3 and Bax; (2) TP53INP1 could displace p62 and form autophagosomes with LC3, which induces autophagy-dependent cell death; (3) TP53INP1 mediated EMT via regulating Smad2/3 signalling. As a TP53INP1 regulator, miR-106a enhances tumour survival and metastasis in different ways: (1) it inhibits TP53INP1 induced autophagy-dependent death; (2) it antagonizes the inhibition role of TP53INP1 on EMT.

The dysregulation of miRNAs has been shown to be the cause in various cancers and is involved in tumour metastasis through regulating diverse biological processes, such as miR-106a [29]. Our data highlighted the mechanism of miR-106a promoting BM of lung adenocarcinoma (Fig. 7). In this study, we demonstrated that miR-106a promoted lung adenocarcinoma BM via directly targeting TP53INP1. In particular, we confirmed that TP53INP1-mediated autophagy-dependent cell death and EMT could be responsible for the pro-metastatic role of miR-106a in lung adenocarcinoma. Given the importance of TP53INP1 in cancer progression, our data revealed the function, mechanism and clinical implication of miR-106a in lung adenocarcinoma with BM.

In a previous study, we found that miR-106a was one of the most pivotal BM-related miRNAs in lung adenocarcinoma by high-throughput sequencing [25]. Tian et al. suggested that NSCLC patients with high expression of miR-106a had a poorer prognosis [21]. In the present study, we found that miR-106a was highly expressed in the clinical samples of lung adenocarcinoma with BM compared to primary lung adenocarcinoma samples. Univariate and multivariate Cox regression analyses indicated that both higher miR-106a expression and BM were adverse prognostic factors indicating higher risk of death by lung adenocarcinoma. Importantly, we confirmed that the upregulation of miR-106a enhanced lung adenocarcinoma metastatic potential in vitro and in vivo, while the downregulation of miR-106a decreased the metastatic potential. MiR-106a belongs to the paralogous miR-106a-363 cluster, which could accelerate tumour development, induce angiogenesis, prevent apoptosis and crucially influence osteoblastic proliferation and differentiation [30]. However, the overexpression or deletion of miR-106a-363 did not affect individual development [31]. This suggested that miR-106a may represent a potential therapeutic target for BM in lung cancer.

Kyoto Encyclopedia of Genes and Genomes (KEGG) pathway analysis revealed that miR-106a regulates DEGs enriched in the p53 signalling pathway. p53 is a well-known tumour suppressor, and p53 mutation contributes to tumorigenesis [32]. The loss or mutations of p53 promotes malignant progression, invasion, metastasis and chemoresistance of tumour cells [33, 34]. Therefore, we directly explored the association between miR-106a and p53 and subsequently investigated the effect of miR-106a on lung adenocarcinoma metastasis. According to KEGG pathway analysis, we selected the p53 network-related DEGs for verification, which also potentially binds to miR-106a. TP53INP1, IGF1 and ZMAT3 were differentially expressed in clinical samples of lung adenocarcinoma with BM, while miR-106a and TP53INP1 showed a significant negative correlation.

Further evidence demonstrated that p53 signalling pathway might play an important role in cancer metastasis by involving miR-106a. In addition, subcellular localization and phosphorylation of p53 were regulated by miR-106a. These results indicated that miR-106a was closely related to the p53 signalling network. TP53INP1 is a tumour suppressor, whose expression is downregulated in different types of cancers from different organs. It was described as a p53 target gene involved in cell death, cell-cycle arrest and cellular migration [35]. Our research showed that TP53INP1 is a target gene of miR-106a and is closely related to the p53 signalling network. Ng et al. showed that TP53INP1 is often selectively downregulated in advanced stage IV and metastatic human hepatocellular carcinoma (HCC) tumours [36]. Furthermore, Liu et al. suggested that miR-155 promotes liver cancer cell EMT and cancer stem cells, in part, by silencing TP53INP1 [37]. In the present study, in vitro and in vivo experiments showed that overexpression of TP53INP1 significantly reversed the promoting effects of miR-106a in the metastatic steps of lung adenocarcinoma, including cell migration, intravasation into the surrounding circulatory system (via EMT) and cell survival. These studies demonstrated the crucial role of miR-106a/TP53INP1 axis in the progression of BM in lung adenocarcinoma.

Furthermore, we examined the effect of miR-106a/TP53INP1 axis on cell death and EMT contributing to tumour cell metastasis. TP53INP1 expression induced a significant increase in late apoptosis and necrosis. Cotransfection with miR-106a mimics resulted in a significant decrease in cell death. Seillier et al. described the TP53INP1 as an inducer of autophagy-dependent cell death rather than an inducer of autophagic cell death [26]. In this study, we first found that miR-106a reversed the autophagy induced by TP53INP1. Then ATG5 knockdown abolished the effect of miR-106a on the number of colonies. Moreover, the pan-caspase inhibitor (Z-VAD-FMK) also suppressed the inhibitory role of miR-106a in cell death induced by TP53INP1 overexpression. These data suggested that miR-106a inhibits TP53INP1-associated autophagy-dependent cell death. Many previous studies indicated that autophagy can help cells resist death [38–40]. However, several recent studies suggested that autophagy contributes to tumour cell death [41–43]. TP53INP1 might induce the anti-proliferative and pro-apoptotic activities. Consistently, we found that several pro-apoptotic proteins [44], such as p21, Bax and Pig3, were shown to be regulated by miR-106a/TP53INP1, suggesting that miR-106a/TP53INP1-modulated autophagy could be a mechanism for tumour suppression. In addition, miR-106a/TP53INP1-regulated EMT demonstrated another mechanism promoting BM of lung cancer.

An increasing number of studies have found that the autophagy ability of tumour cells is a critical factor for cell survival/death, metastasis and activation after dormancy [45]. Our data also showed that miR-106a was involved in TP53INP1-mediated autophagy. However, there are other forms of autophagy that affect cell death, such as autophagic death, and autophagy antagonizes cell death in many tumours. Therefore, we tried to explore the role of autophagy in the process by which miR-106 promotes BM. We found that inhibiting autophagy (knockout of ATG5 and using autophagy inhibitor 3-MA) could partially attenuate the effect of miR-106a on promoting metastasis. This indicated that the effect of miR-106a on promoting BM is partially in a autophagy-dependent manner. Studies on liver cancer have shown that inhibiting autophagy had no obvious effect on the tumour itself but inhibited the remote metastatic ability of the tumour [46]. Knockout of ATG5 and ATG12 can reduce the colonization ability of melanoma cells in the lung and the invasion ability of gliomas, respectively [47, 48]. Our data also revealed that inhibiting autophagy did not affect BM in lung adenocarcinoma but did inhibit the abilities of miR-106a to promote migration and invasion. In vivo experiments also demonstrated that the ability of miR-106a to promote lung adenocarcinoma colonization in the bone was significantly suppressed by inhibiting autophagy. Several studies have shown that miR-106a can inhibit autophagy by targeting and regulating multiple effectors [24, 49, 50].

Finally, we further performed IHC detection of p53, TP53INP1, LC3 and mTOR in tissues of lung adenocarcinoma with or without BM. The results showed that the expression of p53 and TP53INP1 in the lung adenocarcinoma patients with BMs were significantly lower than those of NM patients, but the expression of LC3 and mTOR were not significantly associated with BMs. Because the wild-type p53 protein is rapidly degraded, and the TP53 mutation is usually related to the production of the stable protein that can be detected by IHC in cancer cells, it is generally believed that IHC is ideal for the detection of p53 mutation. However, existing studies [51] have shown that lung cancer cells that have spread rarely accumulate mutated p53 protein, and even if this mutation exists in autologous primary tumours, the derived cell lines do not have p53 mutations. These observations indicated that tumour cells can leave the primary tumour before the p53 gene mutation occurs. Our study also demonstrated that the proportion of negative expression of p53 in lung tissues with BM was much higher than that of NM lung tissues. In addition, we found that miR-106a was negatively correlated with the expression of TP53INP1 and p53, which partially confirmed the results of in vitro cytology, that is, miR-106a inhibited TP53INP1 and/or p53 from participating in BM. Because TP53INP1 interacts with LC3 and ATG8 family proteins through the LC3-interacting region and promotes autophagy-dependent cell death. Therefore, we assessed the expression of LC3 in the metastatic bone tissue and found that it was significantly higher than that in the primary lung tissue. The results of IHC studies in HCC showed that the expression levels of LC3 at the metastatic site were higher than that at the primary site, but autophagy was not activated during the invasion, migration and separation of HCC cells [52]. Our results also did not identify difference in the expression level of LC3 between the lung tissues with BM and NM lung tissues. Similarly, no significant difference was detected in mTOR expression, but its expression in metastatic cancer is higher than that of the primary site, consistent with the existing IHC data [53, 54]. These results suggested a role for autophagy in the metastasis of advanced lung cancer.

Notably, the overexpression of TP53INP1 did not completely reverse the miR-106a’s promoting effects on metastasis, including inhibiting apoptosis and promoting EMT. This may be related to p53 feedback regulation, which has been proved to be a double-edged sword in tumour metastasis. TP53INP1 and p53 have complex mutual regulation. On the one hand, TP53INP1 is a downstream target gene of p53. On the other hand, TP53INP1 induces p53 protein phosphorylation at Ser-46 and activate p53, which is manifested as a positive feedback regulation of p53 [44]. Also, studies have also confirmed that p53 induces expression and/or maturation of several miRNAs, which leads to the repression of critical effector proteins [55, 56]. Although our study identified the role of miR-106a and TP53INP1 in tumour metastasis, the complex interplay between p53–miRNA–TP53INP1 only begin to be explored further. In addition, the role of autophagy in miR-106a-promoted metastasis is inconsistent in vitro and in vivo. Inhibiting autophagy did not obtain the expected additive effect, but miR-106a partially lost promoting metastasis effect. It suggests that miR-106a may also regulate other genes involved in BM, and genes are greatly affected by autophagy. Moreover, whether autophagy, in turn, regulates miR-106a expression needs further exploration.

In summary, based on characterization of single miRNA in the p53 feedback pathway, we should explore more strategies to comprehensively and genome-wide identify p53-regulated miRNAs and their target genes.

## Conclusions

Taken together, our study suggested that miR-106a and its target gene TP53INP1 could regulate cell migration, autophagy-dependent cell death and EMT, affecting lung adenocarcinoma BM. Considering the challenge of targeting therapeutic miRNAs such as miR-106a, the regulation of miRNAs target genes is crucial for preventing metastasis, such as TP53INP1. It is a more amenable potential target for the treatment of advanced NSCLC patients with BM.

## Materials and methods

### Patients and samples

Eighty patients who had primary lung adenocarcinoma and lung adenocarcinoma with BM were enrolled in this study between January 2014 and December 2017 from Yunnan Cancer Hospital, China. Each tissue sample was divided, and half of the tissue was frozen using liquid nitrogen and stored at −80 °C. The remaining tissue was fixed in 10% neutral buffered formalin. Our study was approved by the medical ethics committee of Yunnan Cancer Hospital, China. Prior informed consent was obtained from the patients for collecting tissue specimens and clinical parameters according to the guidelines of Yunnan Cancer Hospital, China. Information regarding the clinicopathological characteristics of 80 lung adenocarcinoma patients is presented in Table S1 and Fig. S3.

### RNA isolation and quantitative real-time PCR

Total RNA was extracted from tissue samples or cells using TRIzol (Invitrogen, USA) following the manufacturer’s protocol. For miRNAs, first-strand cDNA was reverse transcribed using a miRcute Plus miRNA First-Strand cDNA Kit (TianGEN, China), but for mRNAs, a ReverTra Ace qPCR RT Kit (TOYOBO, Shanghai) was used to synthesize first-strand cDNA. Then quantitative real-time PCR was performed using SYBR FAST qPCR Master Mix (KAPA Biosystem, USA) on a Quantstudio 12K FLEX system (Applied Biosystems, USA). GAPDH and U6 were used as internal controls for mRNA and miRNA, respectively. For each sample, all experiments were performed in triplicate. The fold change (FC) in mRNA was calculated according to the relative quantification (2^−ΔΔCt^). The primer sequences used in our study are shown in Table S5.

### Cell lines and transfection

The human lung adenocarcinoma cell lines A549 and SPC-A1 were obtained from Kunming Institute of Zoology and cultured in Dulbecco’s Modified Eagle Medium (DMEM; Invitrogen, USA) supplemented with 10% foetal bovine serum (FBS; HyClone, USA). All cells were incubated in a humidified atmosphere of 5% CO_2_ at 37 °C. MiR-106a mimics/inhibitor and mimic control (NC-mimics) were purchased from RiboBio Co, Ltd. (Guangzhou, China). Human full-length TP53INP1 cDNAs were cloned into pEYFP-C1 vector (Clontech, Japan). Adenovirus vectors that direct the expression of Myc-TP53INP1 (AdMax-Myc-TP53INP1) were constructed using the AdMax system (Microbix Biosystems). The regions corresponding to the full sequence of TP53INP1 mRNA were synthesized and cloned into the adenovirus shuttle vector pHBAd. Both A549 and SPC-A1 cells were transfected with miR-106a-mimics, NC-mimics, TP53INP1-ex or NC-ex using Lipofectamine 2000 reagent (Invitrogen, USA) and infected with AdMax-Myc-TP53INP1 (TP53INP1) or AdMax-Myc (Empty) according to the manufacturer’s protocol. Cells were harvested at 24 h post-transfection and used for further experiments. Luciferase-expressing SPC-A1 cells were established in our laboratory by stable transfection with the pCMV-G Luc 2 plasmid (New England Biolabs, USA). Atg5^−/−^ SPC-A1 cells were established using the CRISPR/Cas9 system (Inovogen, Beijing, China). Exon 2 of Atg5 was selected for the design of the guide RNA. Guide RNA sequence: Atg5: 5’-TGCTTCGAGATGTGTGGTT-3’. Small interfering RNA for ATG5 (5’-GCAACUCUGGAUGGGAUUG-3’) was synthesized by Shuoqing Biotechnology Co., Ltd. (China).

### Cell proliferation assay

A CCK-8 kit (Beyotime, China) was utilized to measure the proliferative abilities of A549 and SPC-A1 cells after transfection with miR-106a mimics (miR-106a) or miR-NC according to the manufacturer’s protocol. Twenty-four hours after transfection, 5 × 10^3^ transfected cells were seeded into 96-well plates with 100 μL of culture medium. The medium was replaced with 100 μL fresh culture media containing 10% CCK-8 at different times (1, 2, 3, 4 and 5 days). The absorbance value at 490 nm was measured using a microplate reader (Multiscan FC, Thermo Scientific).

### Wound healing assay

A549, SPC-A1, Atg5^−/−^ A549 and Atg5^−/−^ SPC-A1 cells were transfected with miR-106a as described above. After seeding these cells into 6-well plates, the culture media was replaced with DMEM containing 0.1% FBS before scratching. Confluent cells were mechanically scarred using a 200 μL pipette tip in the centre of the well. Images were captured every 3 h after scarring. The area of wound healing was quantified using the ImageJ software, and the wound healing rate (%) = (initial wound area − nonhealing area)/initial wound area.

### Transwell assay

Transwell assays were performed to assess the invasive abilities of cells using Corning Matrigel Invasion Chamber inserts (8.0 μM pore size, Corning), which were coated with Matrigel (3.9 μg/mL) on the upper surface of the polycarbonic membrane. A549, SPC-A1, Atg5^−/−^ A549 and Atg5^−/−^ SPC-A1 cells were suspended in serum-free medium and seeded into the upper chamber. The bottom chamber was filled with 600 μL DMEM or RPMI-1640 medium supplemented with 20% FBS. After 24 h incubation, the filter was fixed, stained with 0.1% crystal violet, and photographed. The stained cells exhibiting the abilities to invade and migrate were quantified.

### Cell apoptosis assay

Cells were transfected with miR-106a mimic/miR-NC as previously described. Forty-eight hours after transfection, we measured the proportions of viable and apoptotic cells in different treatment groups using the Annexin V FITC Apoptosis Detection Kit (DOJINDO) according to the manufacturer’s instructions. Briefly, cells were harvested using trypsin and were washed in phosphate-buffered saline (PBS). Then cells were resuspended in Annexin V binding buffer (10 mM HEPES, pH 7.4, 140 mM NaCl, 2.5 mM CaCl_2_) and stained with Annexin V-FITC for 10 min and then with propidium iodide at room temperature for an additional 10 min in the dark. After that, cells were analysed by BD FACSCanto II (BD Biosciences) within 1 h after staining. At least 10,000 cells were collected, and the data were analysed using the FlowJo 7.0 software (Tree Star, San Carlos, CA).

### Dual-luciferase reporter assays

Potential targets of miR-106a were predicted using miRanda (http://www.microrna.org). The TP53INP1 3’-UTR sequences containing the presumed miR-106a-binding sites or mutated binding sites were inserted into the psiCHECK™−2 vector (Promega, Madison, WI, USA). A549 and SPC-A1 cells were cotransfected with psiCHECK-2-TP53INP1-WT or psiCHECK-2-TP53INP1-MUT and miR-106a mimic or miR-NC. Forty-eight hours after transfection, luciferase activity was determined with a Dual-Luciferase Reporter Assay Kit (Promega, Madison, WI, USA) according to the manufacturer’s protocol.

### Western blot

After treatment, cells were washed with PBS and lysed in RIPA buffer (Beyotime Institute of Biotechnology, Haimen, China). The protein concentration of lysates was determined using the BCA assay (Pierce; Thermo Fisher Scientific). Equal amounts of proteins were separated by sodium dodecyl sulfate–polyacrylamide gel electrophoresis and transferred onto a polyvinylidene difluoride (PVDF) membrane (Millipore, Billerica, MA, USA). The membranes were blocked with TBST containing 5% bovine serum albumin and incubated overnight at 4 °C with specific primary antibodies according to a certain ratio. Then the membranes were washed three times with TBST and incubated with an anti-rabbit or anti-mouse IgG secondary antibody for 2 h at 25 °C. The primary antibodies used in this study were as follows: LC3 (#2775, 1:1000, Cell Signalling Technology), TP53INP1 (sc-68919, 1:500, Santa Cruz Biotechnology), β-actin (sc-376421, 1:1000, Santa Cruz Biotechnology), histone H3 (sc-517576, 1:2000, Santa Cruz Biotechnology), p-mTOR (Ser2433) (sc-293133, 1:500, Santa Cruz Biotechnology), mTOR (sc-517464, 1:1000, Santa Cruz Biotechnology), p27 (sc-1641, 1:1000, Santa Cruz Biotechnology), p62 (#8025, 1:1000, Cell Signalling Technology), p53 (sc-126, 1:250, Santa Cruz Biotechnology), phospho-p53 (#2521, 1:200, Cell Signalling Technology), acetyl-p53 (#2525, 1:200, Cell Signalling Technology) p21 (sc-6246, 1:250, Santa Cruz Biotechnology), Bax (sc-7480, 1:500, Santa Cruz Biotechnology), and Pig3 (sc-65227, 1:500, Santa Cruz Biotechnology).

The immunoreactive bands were visualized by Pierce™ ECL Western blotting Substrate (Pierce) and captured using a Minichemi TM610 chemical imaging system. The protein band densities were measured by the Quantity One software (Bio-Rad), and the values were normalized to β-actin.

### In situ hybridization

ISH assays were performed using the miRCURY LNATM microRNA ISH Optimization Kit 5 (FFPE) (Exiqon) to detect the expression of miR-106a according to the manufacturer’s instructions.

Briefly, 5 μm sections were first fixed in 4% formaldehyde for 20 min. Next, sections were treated with Proteinase K for 5 min at 37 °C after dewaxing and rehydration, followed by incubation with hybridization mix for 1 h at room temperature. Then the sections were blocked for 20 min in blocking solution and hybridized with digoxigenin-labelled miR-106a probes at 37 °C for 24 h. Nitroblue tetrazolium and 5-bromo-4-chloro-3-indolyphosphate were used as chromogens. Images were acquired on a Leica DM6000B Microscope (Leica Microsystems, Mannheim, Germany).

### H&E and IHC

At the end of the experiment, mice were euthanized, and hind limbs were collected and fixed in 10% neutral buffered formalin for 48 h, followed by decalcification in 10% EDTA for 2 weeks. Next, bone tissues were embedded in paraffin wax for sectioning (thickness, 3.5 μm) and stained with H&E. The patients’ tissue samples, which were fixed in 10% neutral buffered formalin, were also dewaxed and subjected to IHC staining using LC3II antibody (1:200).

IHC was used to measure the expression of p53, TP53INP1, LC3 and mTOR in the lung and bone tissues of lung adenocarcinoma patients. The intensity, percentage and sublocalization of the IHC staining of each case were recorded. The intensity of TP53INP1, LC3 and mTOR staining was recorded as 0, 1, 2 and 3, indicating negative, weak, moderate and strong staining, respectively. The percentages of TP53INP1-, LC3- and mTOR-positive cells were recorded as 0 (0%), 1 (0–20%), 2 (20–50%) and 3 (>50%), respectively. The results were scored by multiplying the percentage of positive cells (*P*) by the intensity (*I*): Formula: *Q* = *P* × *I*; Maximum = 300 [57]. For the p53 immunostaining score, negative (0) was defined as ≤20% of positive cells, and positive (1) was defined as >20% positive cells.

### Mouse model of lung adenocarcinoma BM

To explore whether miR-106a prevents BM, 5 × 10^6^ luciferase-transfected A549 or SPC-A1 cells were suspended in 0.1 mL of PBS and then injected with a 27-G needle into the left heart ventricle of BALB/c-nu/nu mice (male, 4–6 weeks old, purchased from Beijing Vital River Ltd., China). The animals were housed in a pathogen-free environment under controlled conditions of light and humidity in our animal facility for 4 or 5 weeks. After a week, the mice were randomly divided into 8 groups, 6 mice per group (the sample size is usually based on previous experience in reported research with use of the same animals). Then the cells were treated with injections of 10 nmol miR-106a agomir (miR-106a agomir; GenePharma, Shanghai, China), miR-106a antagomir (miR-106a antagomir; GenePharma, Shanghai, China) or NC via the left heart ventricle 3 times a week for 3 weeks. Mice died in the process or showing tumour masses in the heart and/or in the lung because of improper needle placement were excluded from the study. Bone metastases were analysed by BLI, X-ray and histopathologically confirmed with H&E staining. All evaluations were conducted by the investigators blinded to the treatment allocation. Animal protocols were approved from the Institutional Animal Care and Use Committee at the Kunming Medical University Animal Center conformed to the relevant guidelines and legislations.

### Bioluminescence imaging

At the end of miR-106a agomir or NC treatment, mice were injected with 100 μL of the D-luciferin solution (150 mg/kg) (Gold Biotechnology, St. Louis, MO) intraperitoneally (i.p.) 10 min before imaging. During image acquisition, mice were anaesthetized with 50 mg/kg pentobarbital sodium (i.p.) and then imaged using the Xenogen IVIS Spectrum system (Caliper Life Sciences, USA). Signal intensity in both the left and right hind limbs was quantified as photon flux (photons/s/cm^2^/sr) using the Living Image software 4.2 (Caliper Life Sciences).

### X-ray and micro-CT analysis

At the end of miR-106a agomir or NC treatment, nude mice were anaesthetized and subjected to X-ray radiography in the prone position. Osteolytic lesions were identified as demarcated radiolucent lesions on radiographs. The osteolytic lesion area on X-ray was determined from femurs and tibia and were quantified using the ImageJ software. Moreover, we detected and evaluated the bone lesions by micro-CT via calculating the bone volume/total volume, trabecular number, trabecular thickness and trabecular separation.

### RNA-seq

The TruSeq Total RNA-Seq Library Prep Kit was used to construct the library in accordance with standard Illumina procedures, and sequencing was performed on a HiSeq 2000 platform. The sequencing data were mapped to the reference genome using HISAT2. StringTie was used to assemble the transcripts and quantify gene expression via the FPKM method. edgeR was used to identify DEGs, and the cut-off criteria were |FC| ≥ 1.5 and false discovery rate <0.05.

### Statistical analysis

Experiments were performed in triplicate with a minimum of three independent experiments. All data were analysed using the statistical software GraphPad Prism 7.0 (GRAPH PAD Software Inc., CA, USA). Results of all experiments are depicted as mean ± SD. Comparisons of continuous variables between the two groups were performed with two-tailed *t* tests (normal distribution) or Wilcoxon tests (not normal distribution) or log-rank test (for survival curve analysis). A comparison between three or more groups was analysed using one-way analysis of variance with Bonferroni test. Univariate and multivariate analyses were performed using Cox’s proportional hazard model to evaluate the correlation between miR-106a expression and patient prognosis. A value of *P* < 0.05 was considered statistically significant.

## Supplementary information

Supplementary figure and table

## Data Availability

The data sets used and/or analysed during the current study are available from the corresponding author on reasonable request.

## References

[1] Rajapakse P (2021). An update on survivorship issues in lung cancer patients. World J Oncol.

[2] Yang Z, Su Z, DeWitt JP, Xie L, Chen Y, Li X (2017). Fluvastatin prevents lung adenocarcinoma bone metastasis by triggering autophagy. EBioMedicine..

[3] Wu S, Pan Y, Mao Y, Chen Y, He Y (2021). Current progress and mechanisms of bone metastasis in lung cancer: a narrative review. Transl Lung Cancer Res.

[4] Jin JJ, Xu TT, Li YF, Wang HY, Zhang D, Zhang PP (2020). Effect of the standardized management of cancer pain on patients with bone metastasis of lung cancer in China. Cancer Manag Res.

[5] Prieto-Fernandez E, Aransay AM, Royo F, Gonzalez E, Lozano JJ, Santos-Zorrozua B (2019). A comprehensive study of vesicular and non-vesicular miRNAs from a volume of cerebrospinal fluid compatible with clinical practice. Theranostics..

[6] Zhang X, Sai B, Wang F, Wang L, Wang Y, Zheng L (2019). Hypoxic BMSC-derived exosomal miRNAs promote metastasis of lung cancer cells via STAT3-induced EMT. Mol Cancer.

[7] Bonci D, Coppola V, Patrizii M, Addario A, Cannistraci A, Francescangeli F (2016). A microRNA code for prostate cancer metastasis. Oncogene..

[8] Wa Q, Li L, Lin H, Peng X, Ren D, Huang Y (2018). Downregulation of miR19a3p promotes invasion, migration and bone metastasis via activating TGFbeta signaling in prostate cancer. Oncol Rep.

[9] Huang S, Wa Q, Pan J, Peng X, Ren D, Huang Y (2017). Downregulation of miR-141-3p promotes bone metastasis via activating NF-kappaB signaling in prostate cancer. J Exp Clin Cancer Res.

[10] Taipaleenmaki H, Browne G, Akech J, Zustin J, van Wijnen AJ, Stein JL (2015). Targeting of Runx2 by miR-135 and miR-203 impairs progression of breast cancer and metastatic bone disease. Cancer Res.

[11] Croset M, Pantano F, Kan CWS, Bonnelye E, Descotes F, Alix-Panabieres C (2018). miRNA-30 family members inhibit breast cancer invasion, osteomimicry, and bone destruction by directly targeting multiple bone metastasis-associated genes. Cancer Res.

[12] Johnson RW, Merkel AR, Page JM, Ruppender NS, Guelcher SA, Sterling JA (2014). Wnt signaling induces gene expression of factors associated with bone destruction in lung and breast cancer. Clin Exp Metastasis.

[13] Taipaleenmaki H, Farina NH, van Wijnen AJ, Stein JL, Hesse E, Stein GS (2016). Antagonizing miR-218-5p attenuates Wnt signaling and reduces metastatic bone disease of triple negative breast cancer cells. Oncotarget..

[14] Liu X, Cao M, Palomares M, Wu X, Li A, Yan W (2018). Metastatic breast cancer cells overexpress and secrete miR-218 to regulate type I collagen deposition by osteoblasts. Breast Cancer Res.

[15] Zou P, Zhu M, Lian C, Wang J, Chen Z, Zhang X (2019). miR-192-5p suppresses the progression of lung cancer bone metastasis by targeting TRIM44. Sci Rep.

[16] Kuo PL, Liao SH, Hung JY, Huang MS, Hsu YL (2013). MicroRNA-33a functions as a bone metastasis suppressor in lung cancer by targeting parathyroid hormone related protein. Biochim Biophys Acta.

[17] Gong M, Ma J, Guillemette R, Zhou M, Yang Y, Yang Y (2014). miR-335 inhibits small cell lung cancer bone metastases via IGF-IR and RANKL pathways. Mol Cancer Res.

[18] Pan YJ, Zhuang Y, Zheng JN, Pei DS (2016). MiR-106a: promising biomarker for cancer. Bioorg Med Chem Lett.

[19] Zhu GF, Xu YW, Li J, Niu HL, Ma WX, Xu J (2019). Mir20a/106a-WTX axis regulates RhoGDIa/CDC42 signaling and colon cancer progression. Nat Commun.

[20] Zhao H, Yan P, Wang J, Zhang Y, Zhang M, Wang Z (2019). Clinical significance of tumor miR-21, miR-221, miR-143, and miR-106a as biomarkers in patients with osteosarcoma. Int J Biol Markers.

[21] Ye T, Changyu S, Limeng Z, Yuan P (2018). Clinical significance of miRNA - 106a in non-small cell lung cancer patients who received cisplatin combined with gemcitabine chemotherapy. Cancer Biol Med.

[22] Qin Y, Chen X, Liu Z, Tian X, Huo Z (2018). miR-106a reduces 5-fluorouracil (5-FU) sensitivity of colorectal cancer by targeting dual-specificity phosphatases 2 (DUSP2). Med Sci Monit..

[23] Lim EL, Trinh DL, Ries RE, Wang J, Gerbing RB, Ma Y (2017). MicroRNA expression-based model indicates event-free survival in pediatric acute myeloid leukemia. J Clin Oncol.

[24] Rothschild SI, Gautschi O, Batliner J, Gugger M, Fey MF, Tschan MP (2017). MicroRNA-106a targets autophagy and enhances sensitivity of lung cancer cells to Src inhibitors. Lung Cancer.

[25] Xie L, Yang Z, Li G, Shen L, Xiang X, Liu X (2013). Genome-wide identification of bone metastasis-related microRNAs in lung adenocarcinoma by high-throughput sequencing. PLoS ONE.

[26] Seillier M, Peuget S, Gayet O, Gauthier C, N’Guessan P, Monte M (2012). TP53INP1, a tumor suppressor, interacts with LC3 and ATG8-family proteins through the LC3-interacting region (LIR) and promotes autophagy-dependent cell death. Cell Death Differ.

[27] Hill C, Li J, Liu D, Conforti F, Brereton CJ, Yao L (2019). Autophagy inhibition-mediated epithelial-mesenchymal transition augments local myofibroblast differentiation in pulmonary fibrosis. Cell Death Dis.

[28] Chen HT, Liu H, Mao MJ, Tan Y, Mo XQ, Meng XJ (2019). Crosstalk between autophagy and epithelial-mesenchymal transition and its application in cancer therapy. Mol Cancer.

[29] Rupaimoole R, Slack FJ (2017). MicroRNA therapeutics: towards a new era for the management of cancer and other diseases. Nat Rev Drug Discov.

[30] Manochantr S, Marupanthorn K, Tantrawatpan C, Kheolamai P, Tantikanlayaporn D, Sanguanjit P (2017). The effects of BMP-2, miR-31, miR-106a, and miR-148a on osteogenic differentiation of MSCs derived from amnion in comparison with MSCs derived from the bone marrow. Stem Cells Int.

[31] Ventura A, Young AG, Winslow MM, Lintault L, Meissner A, Erkeland SJ (2008). Targeted deletion reveals essential and overlapping functions of the miR-17 through 92 family of miRNA clusters. Cell..

[32] Fernandez-Antoran D, Piedrafita G, Murai K, Ong SH, Herms A, Frezza C (2019). Outcompeting p53-mutant cells in the normal esophagus by redox manipulation. Cell Stem Cell.

[33] Alexandrova EM, Yallowitz AR, Li D, Xu S, Schulz R, Proia DA (2015). Improving survival by exploiting tumour dependence on stabilized mutant p53 for treatment. Nature..

[34] Wellenstein MD, Coffelt SB, Duits DEM, van Miltenburg MH, Slagter M, de Rink I (2019). Loss of p53 triggers WNT-dependent systemic inflammation to drive breast cancer metastasis. Nature..

[35] Wang X, Wang L, Mo Q, Jia A, Dong Y, Wang G (2016). A positive feedback loop of p53/miR-19/TP53INP1 modulates pancreatic cancer cell proliferation and apoptosis. Oncol Rep.

[36] Ng K-Y, Chan L-H, Chai S, Tong M, Guan X-Y, Lee NP (2017). TP53INP1 downregulation activates a p73-dependent DUSP10/ERK signaling pathway to promote metastasis of hepatocellular carcinoma. Cancer Res.

[37] Liu F, Kong X, Lv L, Gao J (2015). TGF-β1 acts through miR-155 to down-regulate TP53INP1 in promoting epithelial–mesenchymal transition and cancer stem cell phenotypes. Cancer Lett.

[38] Suzuki J, Nakajima W, Suzuki H, Asano Y, Tanaka N (2017). Chaperone-mediated autophagy promotes lung cancer cell survival through selective stabilization of the pro-survival protein, MCL1. Biochem Biophys Res Commun.

[39] Das CK, Mandal M, Kögel D (2018). Pro-survival autophagy and cancer cell resistance to therapy. Cancer Metastasis Rev.

[40] Lee CS, Lee LC, Yuan TL, Chakka S, Fellmann C, Lowe SW (2019). MAP kinase and autophagy pathways cooperate to maintain RAS mutant cancer cell survival. Proc Natl Acad Sci USA.

[41] Yao ZQ, Zhang X, Zhen Y, He XY, Zhao S, Li XF (2018). A novel small-molecule activator of Sirtuin-1 induces autophagic cell death/mitophagy as a potential therapeutic strategy in glioblastoma. Cell Death Dis.

[42] Kim TW, Lee SY, Kim M, Cheon C, Ko SG (2018). Kaempferol induces autophagic cell death via IRE1-JNK-CHOP pathway and inhibition of G9a in gastric cancer cells. Cell Death Dis.

[43] Wong VKW, Zeng W, Chen J, Yao XJ, Leung ELH, Wang QQ (2017). Tetrandrine, an activator of autophagy, induces autophagic cell death via PKC-α inhibition and mTOR-dependent mechanisms. Front Pharmacol.

[44] Shahbazi J, Lock R, Liu T (2013). Tumor protein 53-induced nuclear protein 1 enhances p53 function and represses tumorigenesis. Front Genet.

[45] Guo JY, Chen HY, Mathew R, Fan J, Strohecker AM, Karsli-Uzunbas G (2011). Activated Ras requires autophagy to maintain oxidative metabolism and tumorigenesis. Genes Dev.

[46] Peng YF, Shi YH, Ding ZB, Ke AW, Gu CY, Hui B (2013). Autophagy inhibition suppresses pulmonary metastasis of HCC in mice via impairing anoikis resistance and colonization of HCC cells. Autophagy..

[47] Maes H, Kuchnio A, Peric A, Moens S, Nys K, De Bock K (2014). Tumor vessel normalization by chloroquine independent of autophagy. Cancer Cell.

[48] Macintosh RL, Timpson P, Thorburn J, Anderson KI, Thorburn A, Ryan KM (2012). Inhibition of autophagy impairs tumor cell invasion in an organotypic model. Cell Cycle.

[49] Zhai Z, Wu F, Chuang AY, Kwon JH (2013). miR-106b fine tunes ATG16L1 expression and autophagic activity in intestinal epithelial HCT116 cells. Inflamm Bowel Dis.

[50] Cui X, Wang X, Zhou X, Jia J, Chen H, Zhao W (2020). miR-106a regulates cell proliferation and autophagy by targeting LKB1 in HPV-16-associated cervical cancer. Mol Cancer Res.

[51] Offner S, Schmaus W, Witter K, Baretton GB, Schlimok G, Passlick B (1999). p53 gene mutations are not required for early dissemination of cancer cells. Proc Natl Acad Sci USA.

[52] Peng YF, Shi YH, Shen YH, Ding ZB, Ke AW, Zhou J (2013). Promoting colonization in metastatic HCC cells by modulation of autophagy. PLoS ONE.

[53] Lee H (2017). Phosphorylated mTOR expression profiles in human normal and carcinoma tissues. Dis Markers.

[54] Hager M, Haufe H, Lusuardi L, Schmeller N, Kolbitsch C (2011). PTEN, pAKT, and pmTOR expression and subcellular distribution in primary renal cell carcinomas and their metastases. Cancer Invest.

[55] Soond SM, Kozhevnikova MV, Townsend PA, Zamyatnin AA,Jr (2020). Integrative p53, micro-RNA and cathepsin protease co-regulatory expression networks in cancer. Cancers.

[56] Rokavec M, Li H, Jiang L, Hermeking H (2014). The p53/microRNA connection in gastrointestinal cancer. Clin Exp Gastroenterol.

[57] Charafe-Jauffret E, Tarpin C, Bardou VJ, Bertucci F, Ginestier C, Braud AC (2004). Immunophenotypic analysis of inflammatory breast cancers: identification of an ‘inflammatory signature’. J Pathol.

