# Supplementary material for: MicroRNA-106a regulates autophagy-related cell death and EMT by targeting TP53INP1 in lung cancer with bone metastasis
Source: Cell Death Dis. 2021 Oct 30;12(11):1037. doi: 10.1038/s41419-021-04324-0 (PMC8557209; doi:10.1038/s41419-021-04324-0)
Supplement: Supplementary file 1 — Supplementary figure and table [file 41419_2021_4324_MOESM1_ESM.doc]

**
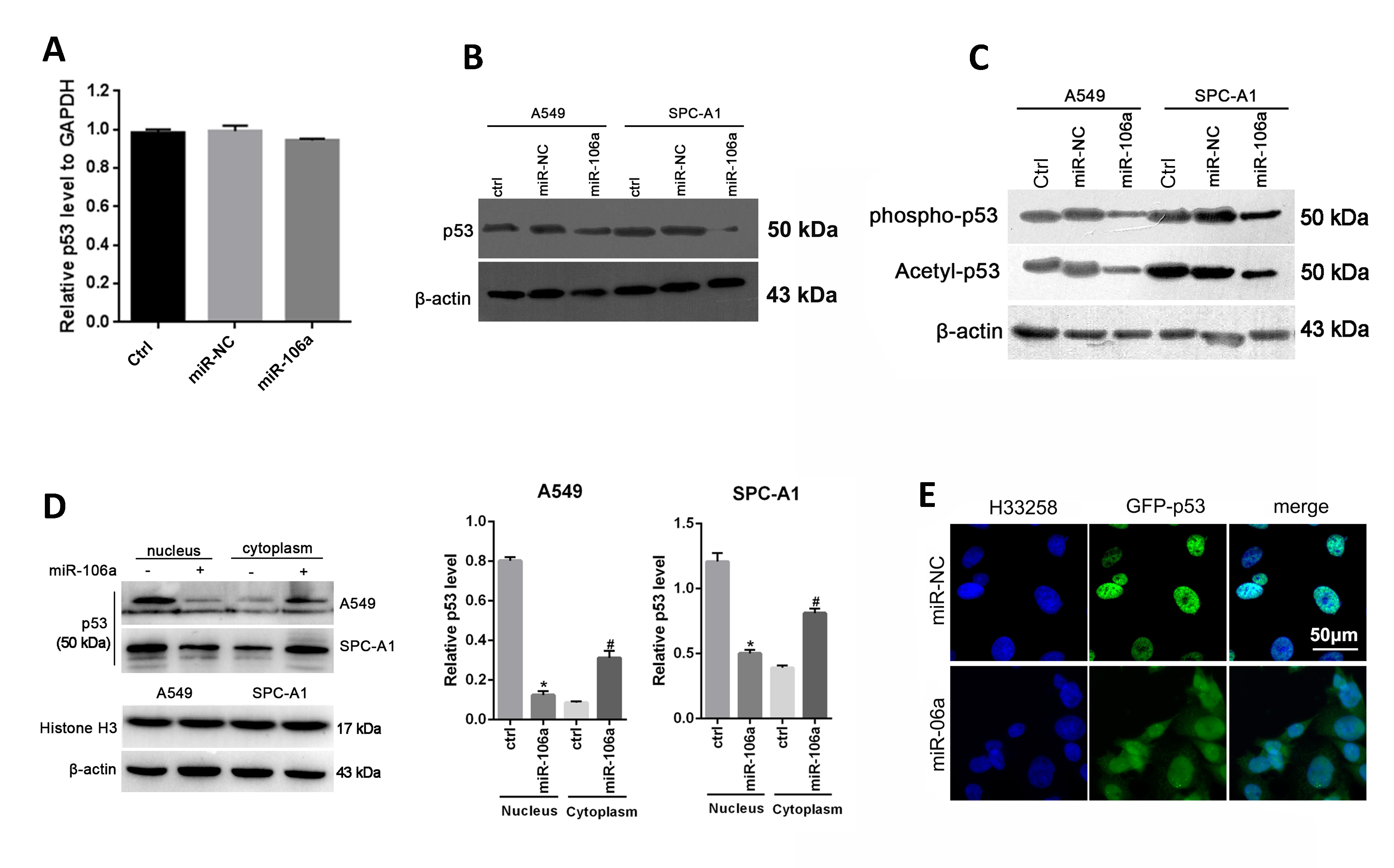
**

**Figure S1. miR-106a regulates the cellular localization and phosphorylation of p53.** After transfected with miR-106a/miR-NC, the expression of p53 at mRNA and protein level was detected by qRT-PCR (A) and Western blot (C). The phosphorylation (Ser46) and acetylation (Lys-382) of p53 was determined by western blot (B). And the cell localization was determined by Western blot (D) and Immunofluorescence (E). Scale bars:50 μm. **P* < 0.05, Nucleus, miR-106a vs. ctrl; ^#^*P* < 0.05, Cytoplasm, miR-106a vs. ctrl.


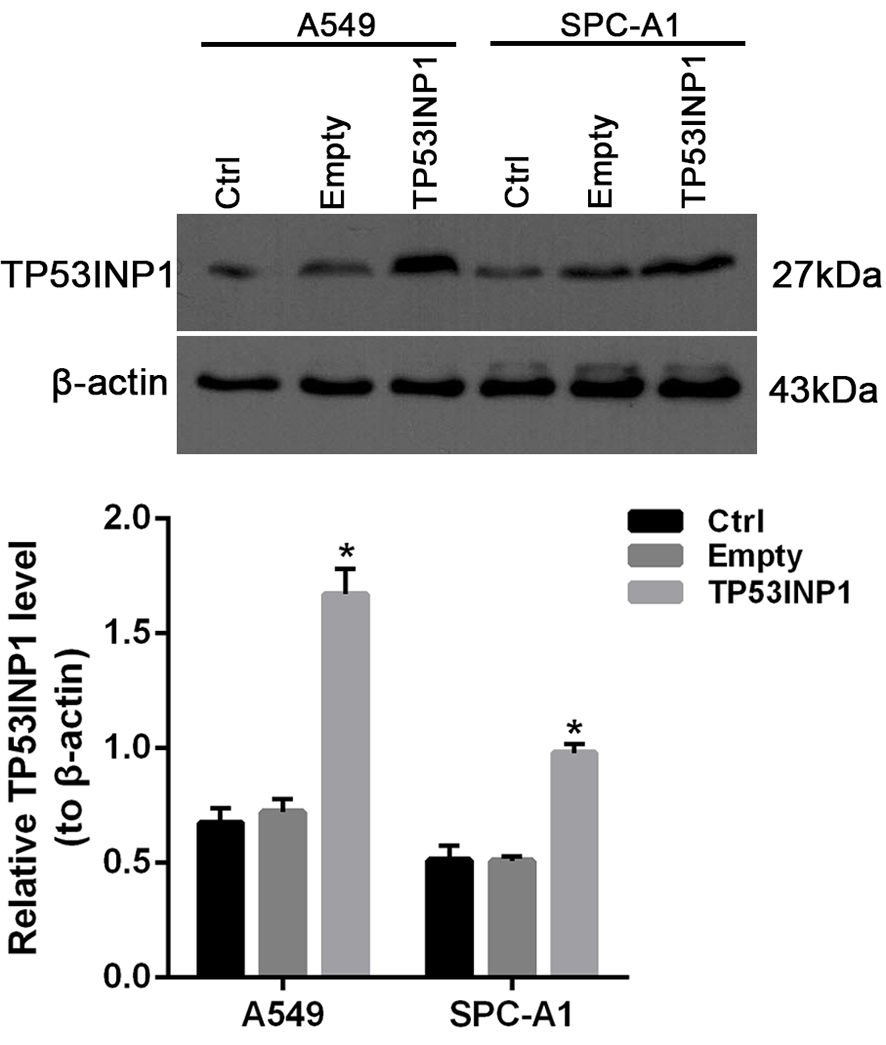


**Figure S2.** **Overexpression of TP53INP1 in A549 and SPC-A1 cells post- transfection was verified by Western-blot analysis.** Empty: vector; TP53INP1: TP53INP1 overexpression plasmid. **P* < 0.05 vs. empty.

**
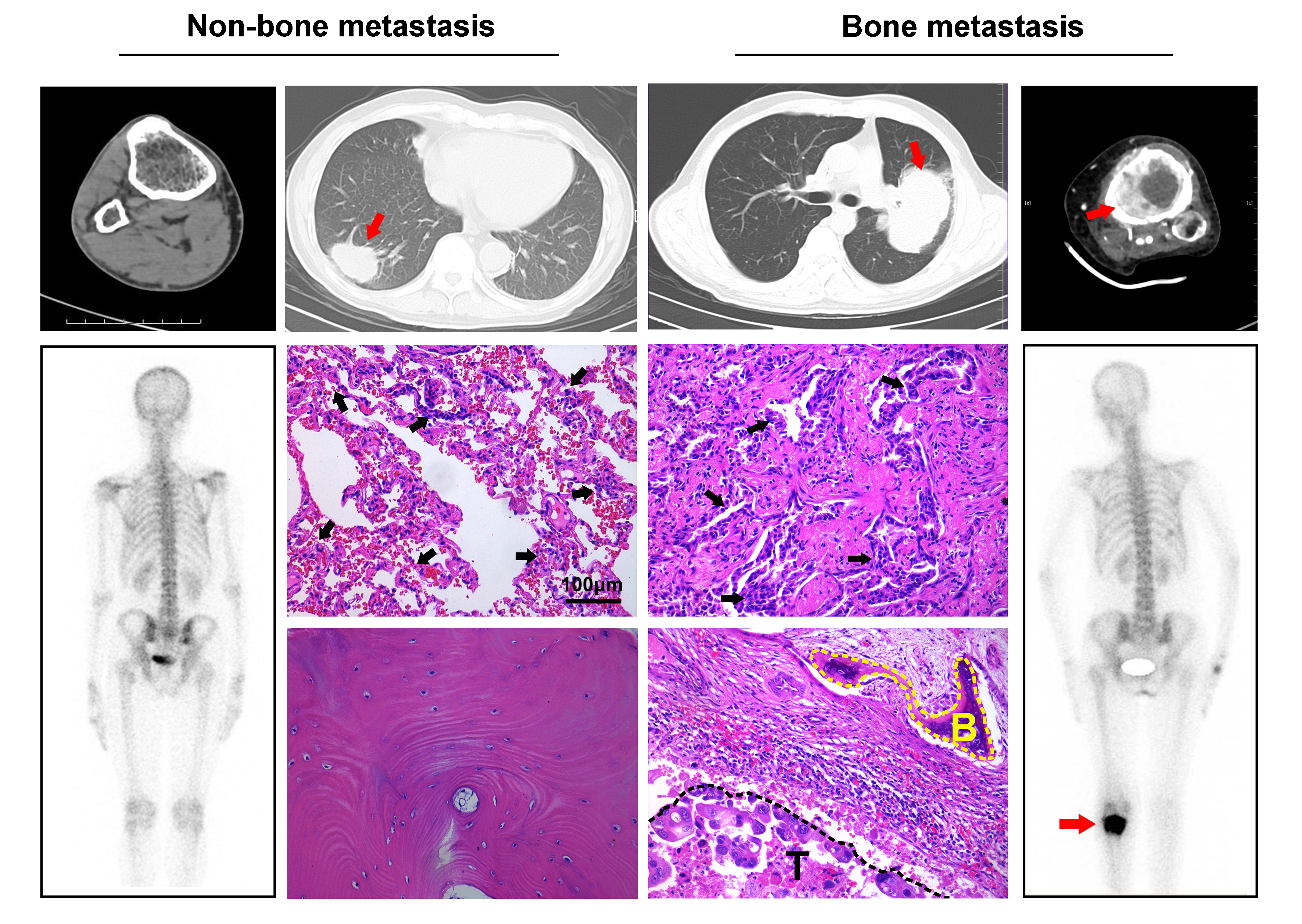
**

**Figure S3.** The differences of lung CT, whole-body bone scan, lung tissue and bone tissue HE stain between the patients with lung adenocarcinoma bone metastasis and non-bone metastasis. Scale bars:100 μm.

**Table S1. Univariate and multivariate analyses of factors associated with overall survival in NSCLC patients.**

| Variable | Univariable model | |  | Multivariable model | |
| --- | --- | --- | --- | --- | --- |
|  | HR(95% CI for HR) | P value |  | HR(95% CI for HR) | P value |
| Gender VS.male | 1.128(0.653,1.946) | 0.666 |  |  |  |
| Age, per year | 1.015(0.985,1.046) | 0.327 |  |  |  |
| Differentiated degree VS. high and middle | 1.587(0.910,2.769) | 0.104 |  |  |  |
| Lymphatic metastasis VS. NO | 1.791(1.001,3.203) | 0.050 |  |  |  |
| Bone metastasis VS. NO | 4.016(2.083,7.774) | 0.000 |  | 2.247(1.008,5.001) | 0.048 |
| MiR-106a expression VS.low* | 4.359(2.334,8.141) | 0.000 |  | 2.735(1.285,5.821) | 0.009 |

**Table S2. The enriched KEGG pathways of miR-106a treatment SPC-A-1 cells.**

| **ID** | **Description** | **GeneRatio** | **pvalue** | **qvalue** | **Gene Name** |
| --- | --- | --- | --- | --- | --- |
| **hsa05322** | Systemic lupus erythematosus | 30/353 | 1.46E-12 | 3.31E-10 | C7/ H2BC14/ H2BC4/ H2AC14/ HIST1H3B/ H2BC10/ H2BC9/ H2AC12/ H3C15/ H3C14/ H2AC17/ H3C7/ HIST1H3C/ H2AC13/ H3C8/ H4C1/ H2BC17/ H2BC13/ H4C14/ H2BC15/ H2AC11/ H3C10/ H2BC6/ H3C11/ H3C4/ CTSG/ H2BC8/ H2BC7/ H4C6/ HLA-DOA/ |
| **hsa05034** | Alcoholism | 35/353 | 2.87E-12 | 3.31E-10 | H2BC14/ H2BC4/ NTRK2/ SHC4/ H2AC14/ HIST1H3B/ H2BC10/ H2BC9/ H2AC12/ H3C15/ H3C14/ H2AC17/ H3C7/ HIST1H3C/ H2AC13/ H3C8/ H4C1/ BDNF/ H2BC17/ MAOA/ FOSB/ H2BC13/ H4C14/ PPP1R1B/ GNG11/ H2BC15/ H2AC11/ H3C10/ H2BC6/ H3C11/ H3C4/ H2BC8/ MAOB/ H2BC7/ H4C6/ |
| **hsa05202** | Transcriptional misregulation in cancers | 24/353 | 2.91E-05 | 0.002153 | IGF1/ NR4A3/ HIST1H3B/ NGFR/ MMP9/ H3C15/ H3C14/ H3C7/ HIST1H3C/ PBX1/ PROM1/ H3C8/ IL6/ ZBTB16/ PAX8/ GZMB/ JUP/ PLAU/ H3C10/ H3C11/ H3C4/ TSPAN7/ ETV7/ SPINT1/ |
| **hsa05410** | Hypertrophic cardiomyopathy (HCM) | 15/353 | 3.74E-05 | 0.002153 | IGF1/ DES/ ACTC1/ DMD/ IL6/ PRKAA2/ ITGA7/ ITGB4/ PRKAG2/ ITGB6/ SGCA/ TPM2/ LAMA2/ TNNT2/ ITGA9/ |
| **hsa04512** | ECM-receptor interaction | 13/353 | 0.000207 | 0.009555 | CD36/ THBS4/ LAMB3/ LAMC2/ SV2B/ COL4A6/ RELN/ ITGA7/ ITGB4/ ITGB6/ LAMA2/ HMMR/ ITGA9/ |
| **hsa04974** | Protein digestion and absorption | 13/353 | 0.00053 | 0.020354 | COL11A1/ COL22A1/ PRSS3/ COL14A1/ SLC3A1/ KCNN4/ COL4A6/ ATP1A2/ SLC15A1/ COL21A1/ FXYD2/ KCNE3/ ACE2/ |
| **hsa05414** | Dilated cardiomyopathy (DCM) | 13/353 | 0.000809 | 0.026645 | IGF1/ DES/ ACTC1/ DMD/ PLN/ ITGA7/ ITGB4/ ITGB6/ SGCA/ TPM2/ LAMA2/ TNNT2/ ITGA9/ |
| **hsa04060** | Cytokine-cytokine receptor interaction | 25/353 | 0.001869 | 0.048737 | CCL14/ CXCL5/ NGFR/ LEPR/ GHR/ CCL20/ CX3CR1/ PDGFD/ LIFR/ TNFRSF11A/ IL6/ CXCL12/ CXCL13/ TSLP/ CXCL10/ CXCL14/ TNFSF4/ EDAR/ IL17RB/ CCL13/ CXCL11/ EDA/ IL21R/ TNFSF15/ HGF/ |
| **hsa05412** | Arrhythmogenic right ventricular cardiomyopathy (ARVC) | 11/353 | 0.001903 | 0.048737 | DES/ DMD/ ITGA7/ ITGB4/ ITGB6/ DSP/ JUP/ SGCA/ CDH2/ LAMA2/ ITGA9/ |
| **hsa04115** | p53 signaling pathway | 10/353 | 0.003082 | 0.071041 | IGF1/ SFN/ TP53INP1/ RRM2/ CD82/ CDK1/ CCNB2/ GADD45B/ ZMAT3/ STEAP3/ |
| **hsa00380** | Tryptophan metabolism | 7/353 | 0.003458 | 0.072468 | AOX1/ INMT/ AOC1/ MAOA/ IDO1/ MAOB/ KYNU/ |
| **hsa00360** | Phenylalanine metabolism | 4/353 | 0.00899 | 0.159867 | PAH/ MAOA/ AOC3/ MAOB/ |
| **hsa05205** | Proteoglycans in cancer | 19/353 | 0.009015 | 0.159867 | IGF1/ WNT2/ MMP9/ WNT2B/ FLNC/ GPC3/ CBLC/ ERBB3/ ESR1/ CAV1/ PLAU/ IHH/ ANK2/ COL21A1/ FGF2/ HPSE2/ MAPK13/ FZD4/ HGF/ |
| **hsa05218** | Melanoma | 9/353 | 0.009789 | 0.161192 | IGF1/ FGF13/ PDGFD/ FGF10/ CDH1/ FGF5/ FGF7/ FGF2/ HGF/ |
| **hsa05144** | Malaria | 7/353 | 0.01081 | 0.166136 | HBB/ SELE/ CD36/ THBS4/ ACKR1/ IL6/ HGF/ |
| **hsa04151** | PI3K-Akt signaling pathway | 27/353 | 0.012816 | 0.184655 | CHRM2/ IGF1/ NGFR/ GHR/ THBS4/ LAMB3/ FGF13/ PDGFD/ FGF10/ IL6/ LAMC2/ COL4A6/ NR4A1/ PRKAA2/ MYB/ GNG11/ RELN/ ITGA7/ ITGB4/ FGFR4/ ITGB6/ FGF5/ FGF7/ LAMA2/ FGF2/ ITGA9/ HGF/ |

**Table S3. The expression level of p53 signal pathway related gene that regulated by miR-106a in SPC-A-1 cells.**

| **Gene_ID** | **Gene_name** | **logFC** | **logCPM** | **PValue** | **FDR** |
| --- | --- | --- | --- | --- | --- |
| **ENSG00000017427** | IGF1 | -3.92913 | 6.35654 | 8.83E-08 | 3.75E-05 |
| **ENSG00000085117** | CD82 | 2.768225 | 4.467274 | 7.76E-05 | 0.003758 |
| **ENSG00000099860** | GADD45B | -2.40219 | 4.869391 | 0.000489 | 0.012822 |
| **ENSG00000106366** | SERPINE1 | -0.99965 | 6.807089 | 0.12989 | 0.515098 |
| **ENSG00000115107** | STEAP3 | 2.033838 | 2.358893 | 0.003143 | 0.048397 |
| **ENSG00000134057** | CCNB1 | 1.920119 | 2.80351 | 0.004993 | 0.06572 |
| **ENSG00000137801** | THBS1 | -1.06696 | 10.52169 | 0.106251 | 0.465562 |
| **ENSG00000146674** | IGFBP3 | 0.59349 | 8.013802 | 0.365452 | 0.806039 |
| **ENSG00000149554** | CHEK1 | 1.317363 | 2.338211 | 0.050432 | 0.30032 |
| **ENSG00000157456** | CCNB2 | 2.721785 | 2.676625 | 0.000118 | 0.00492 |
| **ENSG00000170312** | CDK1 | 2.719023 | 3.502626 | 0.000107 | 0.004591 |
| **ENSG00000171848** | RRM2 | 3.063267 | 3.517402 | 1.70E-05 | 0.001323 |
| **ENSG00000172667** | ZMAT3 | -2.31359 | 5.312564 | 0.000745 | 0.01721 |
| **ENSG00000175305** | CCNE2 | 1.750954 | 1.92449 | 0.010912 | 0.111824 |
| **ENSG00000175793** | SFN | 3.643265 | 3.255041 | 7.14E-07 | 0.000152 |
| **ENSG00000183765** | CHEK2 | 1.084474 | 2.144692 | 0.10595 | 0.46509 |
| **ENSG00000164938** | TP53INP1 | 3.311231 | 2.760804 | 5.17E-06 | 0.000612 |

**Table S4. Correlation analysis the IHC result of p53, TP53INP1, LC3, mTOR between NM and BM or miR-106 high and low.**

| **Variable** | Bone metastasis | | P value | miR-106a expression | | P value |
| --- | --- | --- | --- | --- | --- | --- |
|  | Yes  (n=48) | No  (n=32) |  | Lower  (n=40) | Higher  (n=40) |  |
| **P53** |  |  |  |  |  |  |
| **Negative** | 44 | 4 | <0.001 | 9 | 30 | <0.001 |
| **Positive** | 4 | 28 |  | 31 | 10 |  |
| **TP53INP1** |  |  |  |  |  |  |
| **Negative** | 24 | 6 | 0.001 | 7 | 23 | <0.001 |
| **weak** | 18 | 10 |  | 15 | 13 |  |
| **strong** | 6 | 16 |  | 18 | 4 |  |
| **LC3** |  |  |  |  |  |  |
| **Negative** | 19 | 13 | 0.565 | 18 | 5 | 0.159 |
| **weak** | 17 | 14 |  | 17 | 14 |  |
| **strong** | 12 | 5 |  | 5 | 12 |  |
| **mTOR** |  |  |  |  |  |  |
| **Negative** | 10 | 2 | 0.135 | 6 | 6 | 0.472 |
| **weak** | 18 | 11 |  | 12 | 17 |  |
| **strong** | 20 | 19 |  | 22 | 17 |  |

**Table S5. Primer sequence of qRT-PCR reactions.**

| Primers |  | Sequences |
| --- | --- | --- |
| MiR-106a | Forward | 5'-AAAAGTGCTTACAGTGCAGGTAG-3' |
| U6 | Forward | 5'-GCTTCGGCAGCACATATACTAAAAT-3' |
| GADPH | Forward | 5'-CTTAGCACCCCTGGCCAAG-3' |
| GADPH | Reverse | 5'-GATGTTCTGGAGAGCCCCG-3' |
| TP53 | Forward | 5'-CCTCAGCATCTTATCCGAGTGG-3' |
| TP53 | Reverse | 5'-TGGATGGTGGTACAGTCAGAGC-3' |
| SIRT7 | Forward | 5'-TGGAGTGTGGACACTGCTTCAG-3' |
| SIRT7 | Reverse | 5'-CCGTCACAGTTCTGAGACACCA-3' |
| MDM4 | Forward | 5'-GTATCAGAGCAGTTAGGTGTTGG-3' |
| MDM4 | Reverse | 5'-GACTTAGAGTCCTCCAGGTCATC-3' |
| HIFa | Forward | 5'-TATGAGCCAGAAGAACTTTTAGGC-3' |
| HIFa | Reverse | 5'-CACCTCTTTTGGCAAGCATCCTG-3' |
| TP53INP1 | Forward | TGATGAATGGATTCTTGTTGACTTC |
| TP53INP1 | Reverse | TGAAGGGTGCTCAGTAGGTGAC |
| ZMAT3 | Forward | GCTCTGTGATGCCTCCTTCAGT |
| ZMAT3 | Reverse | TTGACCCAGCTCTGAGGATTCC |
| SNF | Forward | GGCATCAGAAGACCTACGCCTT |
| SNF | Reverse | CTCCATCTCAGCGTCTGTCAGA |
| IGF | Forward | CTCTTCAGTTCGTGTGTGGAGAC |
| IGF | Reverse | CAGCCTCCTTAGATCACAGCTC |
